# Participant Engagement and Adherence to Providing Smartwatch and Patient-Reported Outcome Data: Digital Tracking of Rheumatoid Arthritis Longitudinally (DIGITAL) Real-World Study

**DOI:** 10.2196/44034

**Published:** 2023-11-07

**Authors:** William B Nowell, Jeffrey R Curtis, Hong Zhao, Fenglong Xie, Laura Stradford, David Curtis, Kelly Gavigan, Jessica Boles, Cassie Clinton, Ilya Lipkovich, Shilpa Venkatachalam, Amy Calvin, Virginia S Hayes

**Affiliations:** 1 Global Healthy Living Foundation Upper Nyack, NY United States; 2 University of Alabama at Birmingham Birmingham, AL United States; 3 Kirklin Solutions Hoover, AL United States; 4 C3i Solutions HCL Horsham, PA United States; 5 Eli Lilly and Company Indianapolis, IN United States; 6 Medidata Solutions, Inc New York, NY United States

**Keywords:** real-world evidence, real-world data, patients, rheumatoid arthritis, patient-reported outcomes, patient-generated health data, mobile technology, wearable digital technology, mobile phone

## Abstract

**Background:**

Digital health studies using electronic patient-reported outcomes (ePROs) and wearables bring new challenges, including the need for participants to consistently provide trial data.

**Objective:**

This study aims to characterize the engagement, protocol adherence, and data completeness among participants with rheumatoid arthritis enrolled in the Digital Tracking of Arthritis Longitudinally (DIGITAL) study.

**Methods:**

Participants were invited to participate in this app-based study, which included a 14-day run-in and an 84-day main study. In the run-in period, data were collected via the ArthritisPower mobile app to increase app familiarity and identify the individuals who were motivated to participate. Successful completers of the run-in period were mailed a wearable smartwatch, and automated and manual prompts were sent to participants, reminding them to complete app input or regularly wear and synchronize devices, respectively, during the main study. Study coordinators monitored participant data and contacted participants via email, SMS text messaging, and phone to resolve adherence issues per a priori rules, in which consecutive spans of missing data triggered participant contact. Adherence to data collection during the main study period was defined as providing requested data for >70% of 84 days (daily ePRO, ≥80% daily smartwatch data) or at least 9 of 12 weeks (weekly ePRO).

**Results:**

Of the 470 participants expressing initial interest, 278 (59.1%) completed the run-in period and qualified for the main study. Over the 12-week main study period, 87.4% (243/278) of participants met the definition of adherence to protocol-specified data collection for weekly ePRO, and 57.2% (159/278) did so for daily ePRO. For smartwatch data, 81.7% (227/278) of the participants adhered to the protocol-specified data collection. In total, 52.9% (147/278) of the participants met composite adherence.

**Conclusions:**

Compared with other digital health rheumatoid arthritis studies, a short run-in period appears useful for identifying participants likely to engage in a study that collects data via a mobile app and wearables and gives participants time to acclimate to study requirements. Automated or manual prompts (ie, “It’s time to sync your smartwatch”) may be necessary to optimize adherence. Adherence varies by data collection type (eg, ePRO vs smartwatch data).

**International Registered Report Identifier (IRRID):**

RR2-10.2196/14665

## Introduction

### Background

Technological advances have created new opportunities for the digital and remote collection of patient-generated data either by collecting electronic patient-reported outcomes (ePROs) via internet-based platforms or by passive biometric gathering with wearable devices [1,2]. Compared with typical clinical studies that rely on in-person visits, digital studies using wearable devices and smartphone apps can enable the collection of a greater volume of data with more continuous and granular measurements and may also reduce the need for face-to-face encounters with study staff. These new methods bring both opportunities and challenges to the collection of data for medical research. Among the challenges are uncertainty about how best to activate participants to consistently provide data per a digital study protocol, how to maintain engagement through the study, how best to capture and store data, and what levels of participant attrition or adherence to study protocols can be reasonably expected.

Digital studies on rheumatoid arthritis (RA) and related rheumatic and musculoskeletal diseases (RMDs) have examined patient engagement and protocol adherence, primarily with feasibility studies. The few studies that exist suggest adherence to wearing data-collecting devices such as smartwatches or fitness trackers may be as high as 70% to 90% [3-7], but definitions of adherence differ across these studies, which tend to be short. Attrition rates are high, especially toward the end of study periods, and without established benchmarks (eg, in traditional clinical trials for RA, attrition is typically ≤15%) [8-10], it is difficult to determine the acceptable level of attrition in a study.

Consensus is lacking on the factors and approaches (eg, SMS text messaging, email, phone, or no reminders) most likely to influence participation and optimize data completeness over time. Expected adherence to completion of questionnaire data, such as ePRO measures, collected at regular (eg, daily or weekly) intervals ranges widely, from <20% to >80%, depending on the length of study, frequency of data collection, and intensity of participant intervention implemented by the study team (eg, reminders, in-person discussion of data, and sharing of results) [11-13]. In short, studies on RMDs using digital data collection to date are heterogeneous, making it difficult to compare findings. An examination of approaches that are most promising for engaging participants in the completion of tasks for digital studies and at what level of anticipated adherence is critical for advancing the field.

### Objectives

Building on lessons learned from a prior pilot study in gout where adherence was suboptimal [4], we modified multiple design elements to promote engagement and adherence to the study protocol among patients with RA in a study requiring daily passive (wearing a smartwatch) and active ePRO data collection. Our objectives were to describe important design features taken to optimize patient engagement and minimize data missingness and to characterize protocol adherence and data completeness among participants enrolled in a longitudinal real-world study of the association between actively reported ePROs and passive data collected from wearables in participants with RA.

## Methods

### Ethical Considerations

The Digital Tracking of Arthritis Longitudinally (DIGITAL) study was an ancillary study of the ArthritisPower registry (Advarra Institutional Review Board protocol #00026788) [14,15]. ArthritisPower was launched in 2015 and comprises members with self-reported RMD who have provided informed consent to participate in research studies and provide data via the ArthritisPower app on a smartphone or web-based equivalent. ArthritisPower protects participant data using the industry standards of computer encryption and data security, as described in the registry informed consent form. Members of the ArthritisPower registry who were residents of the United States or US territories and were aged at least 19 years (≥21 for Puerto Rico residents) with a self-reported physician diagnosis of RA (as indicated by survey screening questions) and smartphone access allowing web-based survey completion were eligible to participate. Potential participants were sent email invitations to join the study; invitation emails included a link that directed potential participants to a landing page with complete information about the study and the opportunity to opt-in by completing an addendum to the ArthritisPower informed consent. Nonresponders to the initial email invitation were sent up to 2 email reminders. Participants who completed all activities for the first 4 weeks received a US $25 gift card; those who completed all activities for the first 12 weeks received an additional US $50 gift card as compensation. Participants were able to keep their smartwatch once they received it, regardless of whether they completed the study.

### Participation

After providing informed consent, participants completed a study registration and demographic survey; they were excluded if they provided a negative response to either of 2 items: not currently on a conventional, targeted synthetic, or biological disease-modifying antirheumatic drug, and not currently seeing a rheumatologist. The eligible participants were then directed to the study-specific customization of the ArthritisPower mobile app to complete the ePROs. For at least 10 days of the 14-day run-in period, participants were required to use the app to complete 2 daily single-item pain and fatigue numeric rating scales, requiring less than 1 minute in total and longer weekly sets of ePROs. Participants who successfully completed the run-in were mailed a wearable device (Fitbit Versa smartwatch) and study materials for the main study. The study development and pilot testing were conducted as described in the DIGITAL protocol [14]. This was a truly web-based trial in which there were no in-person study “visits,” and no study coordinators had any preexisting relationship with participants, as might be the case with a traditional in-person clinical study. The Fitbit Versa was chosen as the study-specific device because of its potential acceptability to participants, ability to capture activity and sleep measures, existing data platform that enabled monitoring smartwatch use and facilitated dataflow, and relatively modest cost.

Participation beyond the main study was observed to determine whether participants would continue to use and synchronize the smartwatch on their own without automated or manual prompts. The in-app ePRO workflow ceased for each participant once they concluded the main study; therefore, we did not monitor whether the participants continued to provide ePRO data.

### Monitoring Participant Adherence to the DIGITAL Study Tasks

The main study period included automated and manual prompts to complete ePROs and wear and regularly synchronize the smartwatch. Participants’ progress from registration through the end of the main study period was monitored remotely, and centralized study coordinators contacted participants via email, SMS text messaging, and phone to address and attempt to resolve adherence issues. A priori rules regarding consecutive spans of missing data triggered such participant contacts as needed (Multimedia Appendix 1).

To qualify as having met adherence criteria during the run-in period and qualify for the main study, participants were required to complete at least 1 set of weekly ePRO assessments and 10 (71%) of 14 of the daily ePROs during the run-in period (referred to as “lead-in” throughout the published protocol). This differed slightly from the planned protocol [14] because database programing allowed weekly ePROs to begin on any day of the week, such that individuals could complete their run-in after only 10 days, eliminating the possibility for a second weekly measurement. After participants met these criteria, they were shipped a smartwatch package to begin the main study. Their contact information was recorded in an Access (Microsoft Corporation) database so that the study coordinators could follow-up and provide support as needed. Dates when the smartwatches were shipped and delivered via the US Postal Service and the date on which participants first successfully synchronized their smartwatches were also recorded in the Access database.

The first date on which each participant synchronized their smartwatch for the first time was considered day 0 of the main study for that participant, and by definition, it was <24 hours. Day 1 of the study was defined as the first full 24-hour period that a participant could have contributed smartwatch data. Smartwatch data were captured via Fitabase, a commercial platform that uses the Fitbit Partner application programming interface to provide access to a variety of Fitbit-related data and tools for managing a large number of Fitbit devices, including the Fitbit Versa smartwatch. Participants were asked to charge and synchronize their smartwatches “regularly” but not given a specific timeframe so that we could evaluate how often they would synchronize without prompting. Daily synchronizing was anticipated because the smartwatch would have to be charged about that often to stay powered. On the basis of the storage capacity of the smartwatch, daily synchronizing would also prevent the loss of detailed data that starts occurring at 1 week without synchronizing. Data from the Fitabase and ArthritisPower databases were imported into the Access database to enable monitoring.

The study coordinators monitored study participation daily with the Access database, which was designed to display the completeness of data collection for each participant and highlight any need to identify or correct missing data. In addition to daily checking, the database was programmed to alert study coordinators to predefined gaps in data, and study coordinators responded with predetermined and gradually escalating contacts to participants when those occurred.

The first planned intervention was to send automated messages (via email and lock-screen notifications on the participant’s smartphone) to participants when smartwatch or ePRO data were missing after defined periods (ie, starting 3 d after a participant did not synchronize their watch or submit their ePRO data; Multimedia Appendices 1 and 2). Next, if missing data persisted, study coordinators were to escalate the intervention by having a preformulated SMS text message sent from within the Access database using a Twilio application programming interface (Multimedia Appendix 3). If data lapses persisted despite these automated messages, study coordinators were to reach out to participants by phone. If all predetermined interventions were unsuccessful, a final email was to be sent to the participant asking them to contact the study coordinators to avoid removal from the study because of nonadherence.

If a participant did not respond to an automated or preplanned intervention, the study coordinators could use their discretion to call, SMS text messaging, or email participants to determine and troubleshoot issues with devices or software and other reasons for nonadherence. At the outset of the study, it was assumed that a phone call to the participant from the study coordinator was the highest level of intervention. All automated and study coordinator interventions were logged into the Access database to ensure an accurate record of interactions with the participants. A heat map overview of data completeness was circulated to study leads on a regular basis to keep them informed about study participation and to flag larger issues that required attention. Study coordinators tracked the adherence issues they encountered, along with any identified causes, and any resolution of the issues.

### Statistical Analysis

During the main study, protocol adherence to prespecified data submission was defined as providing (1) all daily ePROs on >59 (70%) of 84 days; (2) all weekly ePROs for at least 9 (75%) of 12 weeks; and (3) daily smartwatch data on >59 (70%) of 84 days, during which participants wore it for at least 1152 of 1440 minutes each day. Composite adherence for the main study was defined as meeting all 3 of the abovementioned adherence definitions.

Frequency summaries were computed to determine data completeness and, therefore, participant adherence to digital tasks during the run-in and main study periods. Frequency analysis was also used to compare the characteristics of participants who did and did not qualify for the main study as well as those who met or did not meet each of the 3 adherence definitions. Statistical significance was set at α=.05 in comparing groups of participants who did or did not qualify for the main study and groups who did or did not meet the adherence definitions. Two-tailed *t* tests were performed for continuous variables, and chi-square tests were performed for categorical variables.

Our choice of 14 days for the run-in period was somewhat arbitrary; however, the Patient Rheumatoid Arthritis Data From the Real World (PARADE) study clearly showed that approximately 50% of attrition occurred within the first 2 weeks of the study [16]. We evaluated whether a lower number of days of adherence or nonadherence would predict whether an individual would meet the criteria for continuing on to the main study (providing ePROs at a rate of 70% or 10 of 14 d). Daily percentage adherence was calculated for each individual on each day of the run-in period by dividing the number of days ePROs were reported by the number of days ePROs could have been reported (equation 1).


Daily percentage adherence = number of days with ePROs recorded / number of days of run-in completed to date (1)

The daily rate of adherence for each individual was then ranked among all individuals who continued to the main study or all who did not. For ease of viewing and comparison, data from every individual in the resulting ranked list of those who provided at least 1 day of run-in data but did not proceed to the main study and data from every sixth individual in the resulting ranked list of those who did proceed to the main study were plotted as a dot with size reflecting the absolute value and color denoting whether the person had achieved a 70% rate (blue for yes and red for no) for each day of the run-in period.

Modeling was also performed to identify which factors were associated with protocol adherence to digital tasks during the main study. A composite measure summarizing high protocol adherence to provide daily ePROs, weekly ePROs, and smartwatch activity data over the 84 days of the study was the main dependent variable of interest. High adherence was defined as providing data for >70% of the 84 study days (ie, ≥59 d for daily ePROs and at least 9 of the 12 wk of weekly ePROs). Penalized logistic regression using Least Absolute Shrinkage and Selection Operator (LASSO) penalty was used to identify factors associated with high protocol adherence [17]. The reported odds ratios (ORs) were based on the unpenalized logistic regression including only factors selected by LASSO. We examined demographics, comorbidities (eg, fibromyalgia), shift-work schedule, adherence with providing at least 10 of 14 daily ePROs during the 14-day run-in period and either of the 2 weekly ePRO batteries, and the scores of each of the daily and ePROs measured during the run-in period as candidate features for the LASSO model. Variable selection was conducted using the “lambdamin + 1SE” criterion (the largest value of penalty that gives the cross-validated loss within 1 SE from the minimum), with bootstrapping used to estimate 95% CIs [18].

## Results

### Participant Recruitment and Demographics

**Figure 1:**
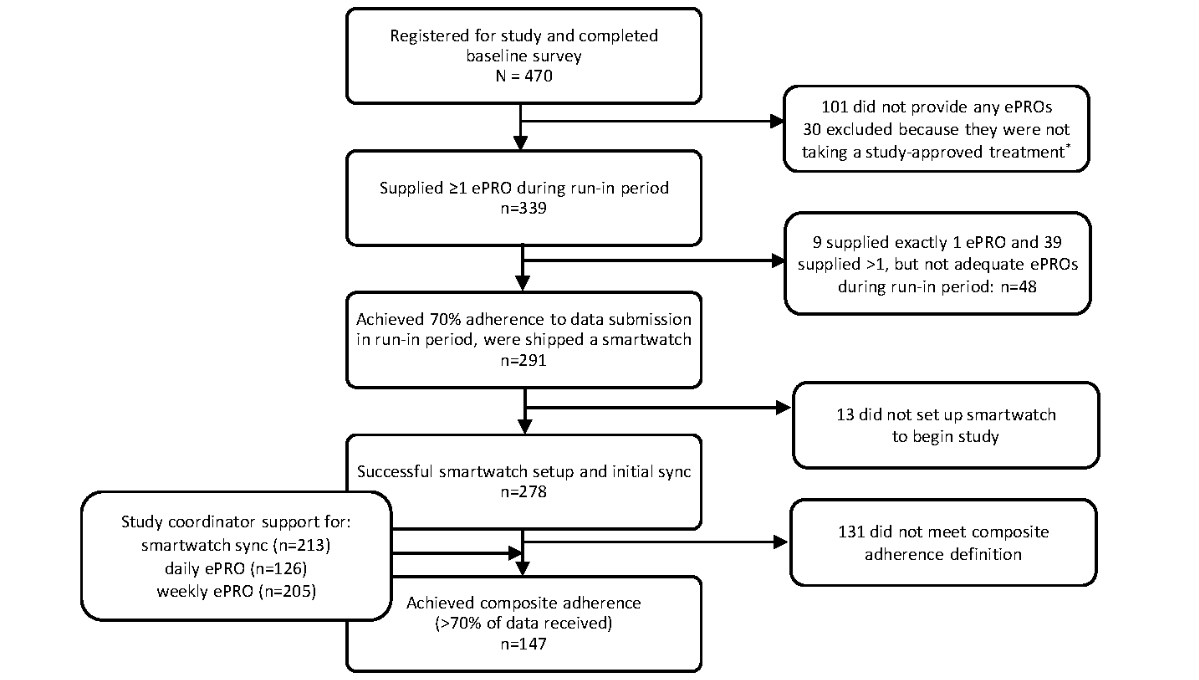
Digital Tracking of Arthritis Longitudinally participant CONSORT (Consolidated Standards of Reporting Trials) flow diagram. *These individuals are all included in Table 1, which shows all data from the baseline surveys. ePRO: electronic patient-reported outcome.

Of the 8772 eligible members of ArthritisPower who were sent emails inviting them to participate from December 23, 2018, to December 10, 2019, a total of 2629 (29.97%) opened the email. Among those who opened the email invitation, 30.77% (809/2629) clicked through to the link to register, and 58.1% (470/809) of those individuals met the inclusion criteria and registered for the study by December 31, 2019 (Figure 1). Of the participants completing registration questions, 61.9% (291/470) qualified for the main study by meeting the definitions of adherence to ePRO data submission and were shipped a smartwatch. Of the 291 participants, 278 (95.5%) set up and synchronized their smartwatch for participation. The 278 participants who qualified for the main study were mostly female (255/278, 91.7%) with a mean age of 50.2 (SD 11.1) years and had received a diagnosis of RA a mean of 9.4 (SD 10.1) years before joining the study (Table 1).

**Table 1:** Demographic and clinical characteristics of participants at baseline and during run-in period, by main study eligibility (n=470).

Characteristics		Met adherence measure in run-in period (n=278)	Did not meet adherence measure in run-in period (n=192)	*P* value
Age (y), mean (SD)		50.20 (11.05)	52.12 (12.09)	.08
Female, n (%)		255 (91.7)	173 (90.1)	.66
White, n (%)		239 (86)	165 (85.9)	.99
Currently employed, n (%)		154 (55.4)	76 (39.6)	.001^a^
Regular daytime work schedule (ie, 9-5; among 130 employed), n (%)		130 (46.8)	65 (33.9)	.001
Years since RA^b^ diagnosis, mean (SD)		9.40 (10.10)	10.51 (10.26)	.25
Osteoarthritis (comorbid), n (%)		124 (44.6)	69 (35.9)	.07
Fibromyalgia (comorbid), n (%)		85 (30.6)	57 (29.7)	.92
Other rheumatic or musculoskeletal condition (comorbid), n (%)		122 (43.9)	92 (47.9)	.44
**Current RA treatment^c^, n (%)**				
	bDMARDs^d^ with or without csDMARDs^e^	176 (63.3)	95 (49.5)	<.001
	tsDMARDS^f^ with or without csDMARDS	34 (12.2)	21 (10.9)	N/A^g^
	csDMARDs without bDMARDs or tsDMARDs	68 (24.5)	55 (28.6)^c^	N/A
	None of the above	0.0	21 (10.9)^h^	N/A
**Daily or weekly PROs^i^ at run-in (baseline), mean (SD)**				
	Pain (daily, 0-10 NRS^j^)	4.9 (2.5)	6.2 (2.5)	<.01^a^
	Fatigue (daily, 0-10 NRS)	5.4 (2.5)	6.8 (2.5)	<.01^a^
	PROMIS^k^ Pain Interference (weekly, T score 0-100)	61.4 (6.6)	63.8 (7.5)	.35
	PROMIS Physical Function (weekly, T score 0-100)	39.2 (6.6)	36.3 (6.3)	.01
	PROMIS Fatigue (weekly, T score 0-100)	60.6 (7.7)	64.5 (8.8)	<.01^a^
	PROMIS Sleep Disturbance (weekly, T score 0-100)	57.2 (7.3)	60.1 (9.2)	.03
	PROMIS Satisfaction with Participation in Discretionary Social Activities (weekly, T score 0-100)	43.3 (6.7)	41.6 (7.6)	.17
	RA Flare (weekly, 0-50)	27.8 (11.3)	33.1 (11.8)	<.01^a^

^a^Statistical significance between groups of patients who qualified and did not qualify for the main study, *P*<.05; 2-tailed *t* tests were performed for continuous variables, and chi-square tests were performed for categorical variables; and *P* values are nominal in nature and should be interpreted in an exploratory manner.

^b^RA: rheumatoid arthritis.

^c^Data included 6 participants who were excluded after answering the baseline survey owing to treatment with a non–study-approved drug.

^d^bDMARDs: biologic disease-modifying antirheumatic drugs.

^e^csDMARDs: conventional synthetic disease-modifying antirheumatic drugs.

^f^tsDMARDs: targeted synthetic disease-modifying antirheumatic drugs.

^g^N/A: not applicable (the *P* value is for the test across current RA treatments not 1 specific current RA treatment).

^h^The 24 people who answered “none of the above” were disqualified as potential participants because they were not taking a study-approved treatment and did not continue into the run-in period or the main study.

^i^PRO: patient-reported outcome.

^j^NRS: numeric rating scale.

^k^PROMIS: Patient-Reported Outcomes Measurement Information System.

### Run-In Period Adherence

Frequency analysis showed that a larger proportion of participants who were adherent to data submission were currently employed (154/278, 55.4% vs 76/192, 39.6%; *P*=.001) and receiving treatment with biologic disease-modifying antirheumatic drugs (176/278, 63.3% vs 95/192, 49.5%; *P*<.001; Table 1). Higher daily pain and fatigue numeric rating scale scores and worse Patient-Reported Outcomes Measurement Information System (PROMIS) physical function, pain interference, fatigue, and satisfaction with social activities scores at baseline correlated with lower rates of data adherence during the run-in period (Table 1). No significant differences in adherence between those who did and did not adhere to data collection in the run-in period were seen with respect to age (*P*=.08), comorbid rheumatic and musculoskeletal conditions (ie, osteoarthritis, *P*=.07; fibromyalgia, *P*=.92; or other, *P*=.44), years since RA diagnosis (*P*=.25), or shift work (*P*=.31).

**Figure 2:**
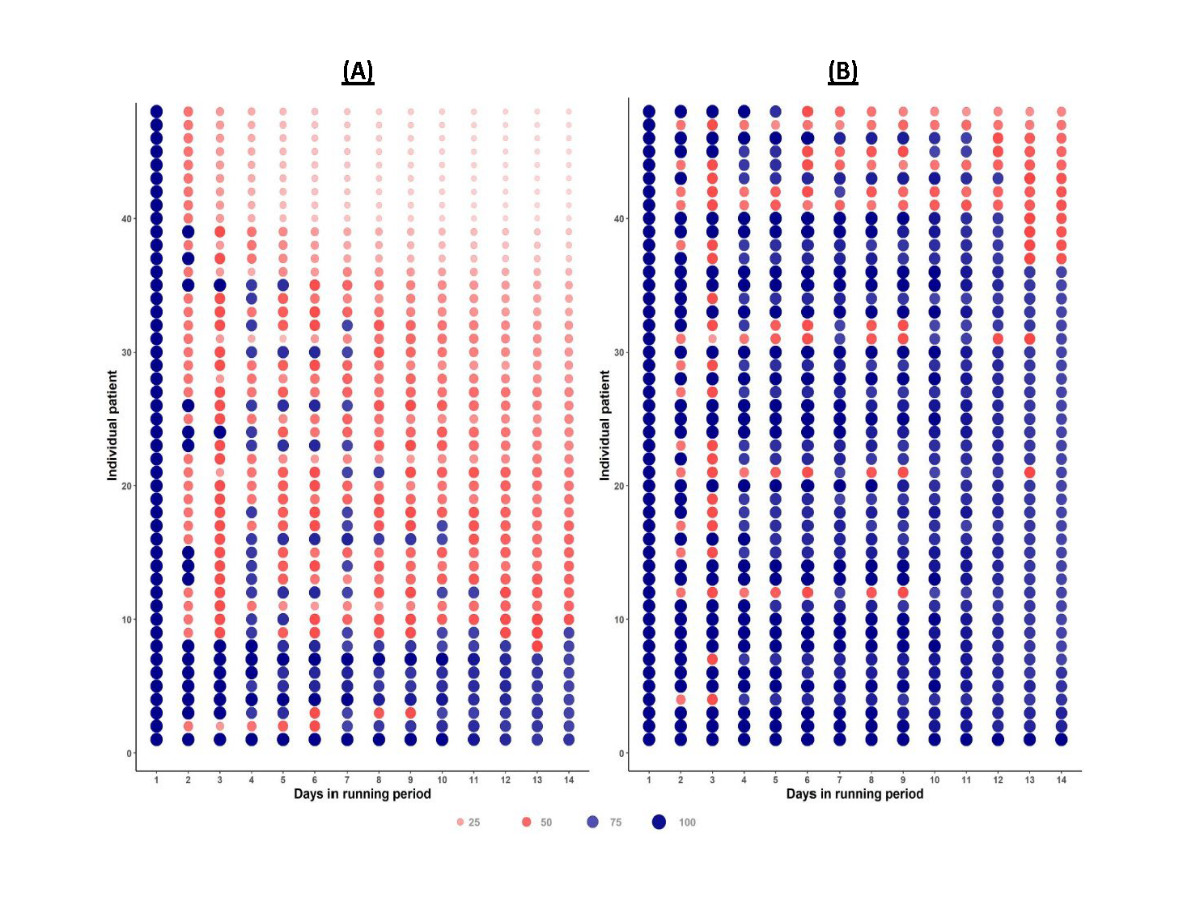
Persistence of electronic patient-reported outcomes data, run-in period. Representative rates of cumulative adherence to data submission show that (A) individuals who did not progress to the main study had a marked drop in adherence as early as day 2; after day 8, few of these participants had the opportunity to qualify for the main study. (B) For those who did progress to the main study, the majority (222/278, 79.9%) were adherent on at least 5 of the first 8 days. Dot size increases with increasing rate of adherence as shown, and at 70% adherence, the color of the dot changes from red (<70% adherence) to blue (>70% adherence). Each dot indicates the representative data from every sixth individual. Rows of all or mostly blue dots at the bottom of plot A and rows of mostly red dots at the top of plot B reflect primarily the individuals who had technical difficulties early in the run-in period who were subsequently allowed to restart the run-in period (accounts for 73% of this artifact) or instances in which the database logged an incorrect date for ePRO submission based on the database change dates on Eastern time rather than the time of the location where the data were logged (14% of artifact), other technical issues such as null scores, manual additions to the database, and duplicate enrollment account for the other 13%. (A) Not included in the main study period and provided >1 day of ePRO run-in data (n=48); each dot represents 1 participant. The dots that are still blue (“adherent”) by day 14 on the left indicate people who qualified but never connected their wearable data or were entered into the main study period after their initial run-in dates had expired. (B) Included in the main study period (n=278), each dot represents every sixth person of the 278 participants who completed the run-in period and qualified for the main study.

Of the 192 registered participants who were eligible for the run-in period but ultimately did not advance to the main study, 48 (25%) provided adequate ePRO run-in data. We generated stacked dot plots to visually compare participants’ persistence in providing ePRO data during the run-in period for people who achieved 70% adherence, set up their smartwatch, and continued into the main study (n=278) versus those who did not (n=48; Figure 2). Among the individuals who did not proceed to the main study, there was a noticeable decline in adherence, starting as early as day 2 for that particular group. By day 8, very few participants remained who even had the opportunity to complete the run-in period successfully. Most participants who did not advance to the main study had low adherence owing to the lack of data reporting by the second or third day of the run-in period.

### Main Study Adherence

Over the 12-week main study period, 52.9% (147/278) of the participants met the predefined composite adherence by providing all 3 types of data submission (ie, daily ePRO submission, weekly ePRO submission, and smartwatch data; Table 2). For the individual components, adherence was highest for weekly ePRO submission at 87.4% (243/278), followed by smartwatch data at 81.7% (227/278) and daily ePRO submission at 57.2% (159/278).

**Table 2:** Completeness of data in the run-in and main study period, by data type (n=278).

Data type	Adherence (n=247), n (%)
Weekly ePROs^a^ ≥9:12 (yes or no)—all PROs^b^ completed	243 (98.4)
Activity data^c^ ≥59:84 d—provided ≥80% of synchronized activity data (1440 min/d)	227 (91.9)
Daily ePROs ≥59:84 d—all PROs completed	159 (64.4)
Composite adherence—daily and weekly ePROs and activity data provided	147 (59.5)

^a^ePRO: electronic patient-reported outcome.

^b^PRO: patient-reported outcome.

^c^Activity data: smartwatch wearable data ≥80% (1440 min/d).

Individuals who met the composite adherence measure were more frequently White and a mean 3.7 (SD 0.72) years older than those who met <3 of the adherence measures (Table 3). No statistically significant association with composite adherence was observed for other baseline characteristics, including comorbid RMD (*P*>.99) or treatment type (*P*=.88), employment status (*P*=.23), pain (*P*=.55), fatigue (*P*=.38), or PROMIS measures evaluated during the run-in period (PROMIS Pain Interference [*P*=.30], PROMIS Physical Function [*P*=.47], PROMIS Fatigue [*P*=.30], PROMIS Sleep Disturbance [*P*=.72], and PROMIS Satisfaction with Participation in Discretionary Social Activities [*P*=.21]). Of the 131 participants who met some, but not all, adherence measures, 80 (61.1%) met smartwatch but not ePRO adherence and 24 (18.3%) met ePRO but not smartwatch adherence. The remaining 20.6% (27/131) of participants who did not meet composite adherence met neither the ePRO nor smartwatch adherence measures.

**Table 3:** Demographic and clinical characteristics of the main study participants at baseline and during the run-in period, by whether composite adherence was met during the main study period (n=278).

Characteristics			Met composite adherence (n=147)		Did not meet composite adherence^a^									Met composite adherence vs pooled did not, *P* value
					Did not meet either activity or PRO^b^ adherence (n=27)		Met activity but not PRO adherence (n=80)		Did not meet activity but did meet PRO adherence (n=24)		Pooled did not meet composite adherence (n=131)			
Age (y), mean (SD)			51.93 (0.57)		43.30 (12.92)		48.56 (10.50)		52.92 (10.06)		48.27 (11.29)		<.01^c^	
Female, n (%)			132 (89.8)		27 (100)		73 (91.2)		23 (95.8)		123 (93.9)		.31	
White, n (%)			133 (90.5)		16 (59.3)		70 (87.5)		20 (83.3)		106 (80.9)		.03^c^	
Currently employed, n (%)			76 (51.7)		17 (63)		52 (65)		9 (37.5)		78 (59.5)		.23	
Regular daytime work schedule (ie, 9-5; among employed), n (%)			64 (43.5)		12 (44.4)		47 (58.8)		7 (29.2)		66 (50.4)		.31	
Years since RA^d^ diagnosis, mean (SD)			9.86 (11.03)		7.74 (8.74)		8.71 (8.72)		10.71 (10.11)		8.88 (8.97)		.42	
Osteoarthritis (comorbid), n (%)			68 (46.3)		10 (37)		35 (43.8)		11 (45.8)		56 (42.7)		.64	
Fibromyalgia (comorbid), n (%)			41 (27.9)		5 (18.5)		32 (40)		7 (29.2)		44 (33.6)		.37	
Other rheumatic or musculoskeletal comorbid condition, n (%)			65 (44.2)		16 (59.3)		31 (38.8)		10 (41.7)		57 (43.5)		>.99	
**Current RA treatment, n (%)**														
	bDMARDs^e^ with or without csDMARDs^f^	95 (64.6)		21 (77.8)		49 (61.3)		11 (45.8)		151 (61.8)		.88		
	tsDMARDS^g^ with or without csDMARDS	35 (23.8)		3 (11.1)		11 (13.8)		3 (12.5)		17 (25.2)		N/A^h^		
	csDMARDs without bDMARDs or tsDMARDs	17 (11.6)		3 (11.1)		20 (25)		10 (41.7)		33 (13)		N/A		
**Run-in daily ePROs^i^, mean (SD)**														
	Pain (0-10 NRS^j^)	4.9 (2.4)		4.6 (2.5)		5.0 (2.6)		5.4 (2.6)		5.0 (2.6)		.55		
	Fatigue (0-10 NRS)	5.3 (2.4)		5.1 (2.7)		5.5 (2.5)		5.6 (2.8)		5.4 (2.6)		.38		
**Run-in weekly ePROs, mean (SD)**														
	PROMIS^k^ Pain Interference (0-100)	61.1 (6.3)		61.5 (7.6)		60.9 (6.1)		64.8 (8.2)		61.8 (7.0)		.30		
	PROMIS Physical Function (0-100)	39.4 (6.8)		39.9 (7.0)		39.3 (5.8)		36.8 (7.3)		39.0 (6.4)		.47		
	PROMIS Fatigue (0-100)	60.2 (7.5)		60.4 (10.0)		61.0 (7.0)		61.5 (8.0)		61.0 (7.9)		.30		
	PROMIS Sleep Disturbance (0-100)	57.0 (6.9)		58.2 (9.3)		56.9 (6.6)		57.5 (10.1)		57.3 (7.9)		.72		
	PROMIS Satisfaction with Participation in Discretionary Social Activities (0-100)	43.6 (6.8)		42.7 (7.4)		43.1 (5.9)		42.1 (7.4)		42.9 (6.5)		.21		
	RA Flare (0-50)	27.1 (11.0)		27.8 (11.8)		27.6 (11.3)		33.5 (11.4)		28.7 (11.6)		.12		

^a^Activity data: smartwatch wearable data≥80% (1440 min/d).

^b^PRO: patient-reported outcome.

^c^Statistical significance between groups of participants who qualified and did not qualify for the main study, *P*<.05; 2-tailed *t* tests were performed for continuous variables, and chi-square tests were performed for categorical variables; and *P* values are nominal in nature and should be interpreted in an exploratory manner.

^d^RA: rheumatoid arthritis.

^e^bDMARDs: biologic disease-modifying antirheumatic drugs.

^f^csDMARDs: conventional synthetic disease-modifying antirheumatic drugs.

^g^tsDMARDs: targeted synthetic disease-modifying antirheumatic drugs.

^h^N/A: not applicable (the *P* value is for the test across current RA treatments not 1 specific current RA treatment).

^i^ePRO: electronic patient-reported outcome.

^j^NRS: numeric rating scale.

^k^PROMIS: Patient-Reported Outcomes Measurement Information System.

The factors associated with high protocol adherence over the 84 days (12 wk) of the main study included age (odds ratio [OR] 1.18/5-y increments, 95% CI 1.06-1.33), high adherence to daily ePROs (completing 10 of the first 14 d; OR 1.73, 95% CI 0.97-3.17), and weekly ePRO adherence during the run-in period (OR 5.31, 95% CI 1.27-36.19). These factors were selected using the LASSO model and strongly associated with high protocol adherence. Additional features included the most recent PROMIS fatigue score before the start of the main study (OR 0.92, 95% CI 0.65-1.3) and the Outcome Measures in Rheumatoid Arthritis Clinical Trials RA Flare score (OR 0.89, 95% CI 0.63-1.26/1 unit change).

The most common time of day to provide ePRO data was morning, in the hours around 10 AM in a participant’s local time zone, when automated app and email notifications were scheduled. Of 23,352 possible person days among 278 participants in the 84-day main study, we observed 19,537 (83.66%) days on which smartwatch activity data were provided for at least 80.0% of the 24-hour period.

The study coordinators contacted participants according to missing data triggers (Multimedia Appendix 3). The most common issue was a participant not synchronizing their smartwatch; this occurred among 76.6% (213/278) of the participants in the main study, followed by issues with weekly (205/278, 73.7%) and daily (126/278, 45.3%) ePRO adherence (Multimedia Appendix 4). A total of 13 participants who were sent smartwatches never synchronized them and therefore never provided activity data. Observations beyond the main study showed that smartwatch use declined by 81% in the first week after the conclusion of the main study period when automated or manual prompts were halted, and no further compensation was expected.

## Discussion

### Recruitment

The recruitment rate for this longitudinal study, which required participants to complete daily and weekly digital tasks over a period of ≥3 months, yielded participant uptake that was similar to other ArthritisPower studies, with comparable invitation email open and click rates. Approximately one-third (2629/8772, 29.97%) of ArthritisPower registry members who were sent emails inviting them to participate opened the email, and 30.77% (809/2629) of those who did so at least began the study registration process. A little over half (470/809, 58.1%) of those who met the eligibility criteria completed the registration. Ultimately, more than one-third (278/809, 34.4%) of those who began registration were able to fully register, satisfy run-in requirements, and start participation in the main study. Attrition at registration should, therefore, be taken into account for the recruitment plans of digital studies with web-based registries such as this one.

### Retention and Adherence

In this digital study with no in-person visits or contact, we found that a 2-week run-in period was more than sufficient to identify the approximately 38.1% (179/470) of participants who would not ultimately reach a 70% level of adherence to planned ePRO submission in the run-in period. Among those who did reach the 70% adherence level and continued into the 12-week main study period, there was a 96% retention rate, with retention defined as submitting any data. For the 3 adherence measures in the main study period, 51.7% (243/470) were adherent to weekly ePRO submission, 48.3% (227/470) were adherent to smartwatch data recording and synchronizing, and 33.8% (159/470) were adherent to daily ePRO submission. These levels of adherence and retention were accomplished using a variety of prespecified escalating strategies for engaging participants and promoting data submission. We used real-time monitoring of data submission adherence and addressed gaps in data first with completely automated approaches that increased to semiautomated and scripted SMS text messages at predefined intervals, and if that did not re-engage a participant, we escalated to custom SMS text messages, emails, and phone calls, as needed. Although data were not available to determine what proportion of participants with missing data re-engaged after each escalating reminder step, very few participants continued the digital task of using and synchronizing their smartwatch on their own (and without our automated and manual prompts) after they completed the main study period.

### Comparison With Prior Work

In traditional clinical trials in participants with RMD where treatment is provided, retention rates of 85% to 90% are typically expected [19,20]. In contrast, web-based studies using wearable devices or smartphones to gather data actively, passively, or both, and where therapy is not provided, have retention rates between 11% and 90%, although the definitions of retention vary widely across these studies [3-5,11,16]. Retention rates are higher when an active reminder system is in place, as in this study, or when only passive data are being collected. A meta-analysis of 10 studies in which participants wore activity trackers showed a mean retention rate of 90% (SD 11%) in studies with a mean cohort size of 34 and mean duration of 10 (range 2-14) wk, similar to the 96% observed in our cohort of 278 over 12 weeks. However, studies in the meta-analysis included systems of reminders with adherence as a secondary measure, but unlike ours, all were intended to improve physical activity. This clear focus on a specific purpose may have boosted adherence, especially because individuals who were not interested in wearing a device to track physical activity may not have agreed to participate [3]. In a longer, 24-week study, there was an 82% retention of 33 individuals with gout who were asked to wear a smartwatch, except when bathing themselves or charging the device. In this study, there were no reminders, but only passive data collection occurred [4]. Other studies, especially those with ePROs or other active data submission but no planned reminder systems, had much lower retention rates. For example, the PARADE study collected ePROs and both active and passive digital data through a customized Apple ResearchKit application used on participants’ chosen devices in a “bring-your-own device” (BYOD) model. The participants received no reminders, although half of the group was randomly assigned to receive reports of their own data at regular intervals. Over 12 weeks, the retention rate among 399 participants was only 11% and did not differ between those who did and did not receive reports of their data [11,16]. We did not evaluate the effect of receiving data reports in this study because the app we used automatically made some ePRO data available to all participants, and participants could monitor their own smartwatch data if they chose to do so.

As a general feature of studies incorporating wearable devices, a BYOD model has both pros and cons to consider. On the positive side, participants already wearing a preferred device need not be trained on how to connect, use it, and interpret its data, lessening the technical support that must be provided at the time of setup. Individuals who have personally chosen a preferred device are more likely to have better adherence to wearing it because it aligns with their own choice. Moreover, those who already have a preferred device are unlikely to want to wear 2—both their preferred device and a study-specific device. Therefore, non-BYOD studies may face greater recruitment challenges. Conversely, requiring a study-specific device allows for homogeneity of the data stream, facilitating analysis. Requiring a study-specific device also allows for better standardization of prompts (eg, “you should charge your device every 4-5 days”) and avoids problems where devices that must be charged regularly (eg, daily, like older Apple Watch models) and are not feasible to capture sleep information.

Definitions and rates of adherence to wearable device data submission vary considerably, from <20% to >80%, depending on the study length and design, including the use of reminders and in-person consultations. Thresholds have been characterized by a minimum number of minutes of usable data per day, for a specified number of days per week, or a percentage of the total study days. In the meta-analysis discussed, only 4 of the 10 studies evaluated adherence [3]. There was a mean rate of 92.7% (SD 4.6%) to wearing a wrist-worn device for a mean duration of 10 weeks across these studies, all of which included reminders in the study design. In the fourth study, there was adherence of 63% to wearing a hip-worn device 80% of the time [3]. In the study of 33 people with gout asked to wear a Fitbit Charge HR2 nearly continuously, 82% provided data for at least 80% of the 1440 possible minutes in a day on 60.5% of the total study days [4]. On the basis of the definition of adherence used in that study, 75% of the participants adhered to a prespecified wear time without reminders [4].

Adherence to ePRO submission is also highly variable, again with differences that appear related to study length and design, including the use of reminders [11-13,16]. In the PARADE study, in which participants provided ePROs via an app installed on their own devices, fewer than half (40.6%) completed ≥1 study assessments as early as the second week of the 12-week study [11,16]. In the Remote Monitoring of RA smartphone app study, 20 participants with RA were asked to provide daily ePROs via the app, which were then imported into electronic health records and discussed during in-clinic consultations. Although daily scores were submitted at a high rate (median 91%, IQR 78%-95% of days), 20% of participants provided scores on >60% of the 90 days of the study [12]. In contrast, in a 4-week study, healthy volunteers and individuals with RA, psoriatic arthritis, or osteoarthritis (n=45) received regular reminders via the data submission app, and 88.3% of ePRO questionnaires were completed overall. Peaks in data submission were observed in the minutes immediately after the automated reminders were sent [13].

### Characteristics of Participants With High Adherence

The rates of retention and adherence in this study more closely matched the rates seen in studies that used wrist-wearable devices, rather than at the hip or on a phone, and also included a regular system of reminders. Unique to this study was the run-in period, which provided the opportunity to habituate participants to data submission and reminders and to select participants who were more likely to be adherent beyond this period. We found that we could identify which participants were not likely to adhere to data submission as early as day 2 of the run-in period in most cases, suggesting that the arbitrarily selected 14 days was longer than needed and that a run-in length of ≤8 days may be optimal.

We also explored whether certain characteristics made it more likely that a participant would complete the study with high rates of protocol adherence and found that people who were employed and using biologic disease-modifying antirheumatic drugs were more likely to complete the 2-week run-in with ≥70% adherence. In contrast, among those who did not qualify, more participants indicated a higher symptom severity. This suggests that there could be a trade-off between high adherence and symptom or disability severity or that participants with higher disability or symptom severity may need relatively more intervention from the study personnel. These findings warrant further exploration so that remote, web-based studies can be planned in a manner that meets the need for a diverse population with sufficient number of participants adhering to data submission.

Considering the factors identified with LASSO analysis as being potentially predictive of adherence, weekly and daily ePRO adherence in the run-in period can be attributed to the study design that intentionally selected people adherent in the run-in period for participation in the main study. We found that such adherence could be identified as early as the second or third day of the run-in period, suggesting that short run-ins are effective. Other factors associated with adherence in the main study were age, associated with higher adherence, and symptom severity, associated with lower adherence.

### Lessons for Remote, Web-Based Study Design With ePROs

This study differed from prior digital studies using wearable devices in several important ways, in that it was larger and entirely web-based with no in-person visits to enable participants to be trained by study staff. Prior studies were small feasibility studies. This study also used a run-in period to habituate participants to providing data and to identify participants who were likely to provide at least minimum levels of data submission. This also minimized the loss of unused smartwatches and reduced the study staff workload related to follow-up on nonadherent participants and missing smartwatches. We also used real-time monitoring of data lapses to modify reminder methods, customizing them to the individual and allowing voice calls, SMS text reminders, and emails as needed. Moreover, data were not used to evaluate healthy behavior change or symptom tracking over time to inform patients’ visit with their physician. Adherence rates were similar to other studies using wearables and reminders to collect passive data and higher than what has been observed in other studies that included active ePRO submission. There are a number of potential reasons for the high level of adherence despite the entirely remote onboarding process and conduct of the study. These include the patient-centric design of materials for the study, the run-in period, and active monitoring by study coordinators with in-app notifications, SMS text reminders, emails, and calls as needed. On the basis of these features, some promising practices for engaging participants in digital studies can be gleaned from this study (Table 4).

**Table 4:** Promising practices for participant retention in digital studies.

Promising practices		Tools for implementation
**Run-in period**		
	Implementing a run-in period habituates participants to providing digital data and allows researchers to exclude individuals likely to be nonadherent and does not need to add more >8 d to the length of a study	In-app prompts Data monitoring
**Compensation**		
	Deferred compensation until after the run-in period allows participants to demonstrate their commitment to participating before taking on additional digital tasks (ie, setting up, wearing, and synchronizing a wearable device) and may optimize overall adherence to the study protocol	Data monitoring Study coordinator
**Automated prompts and “human touch” case management**		
	Considering that study coordinators needed to engage with 90% (n/N) of participants to resolve technical issues in addition to communicating with participants when data lapse occurred, we can infer that the role of study coordinators is an essential part of ensuring adherence in a remote, web-based study. The “human touch” may still be needed even when all data collection is web-based. Anecdotally, study coordinators found that participants were more likely to respond to emails than to phone calls. Rules or triggers and actions in future studies should preference email communication over phone calls to prompt participants	In-app prompts Data monitoring Study coordinator

Given participants’ variable adherence to the completion of digital tasks throughout the study period along with the sharp decline in participation following the main study period, participant support appears to be essential throughout the course of digital studies to optimize quality participant engagement with the study protocol. “Support” in this sense can take many forms, including increased literacy of the applications (ePRO and wearable), familiarity with and ease of use of devices (smartphone and wearable) [21,22], participant satisfaction with the experience (data collection schedules and guidance), and interaction with clinical and study personnel [21,23]. A critical question that still needs to be answered to help improve participant adherence in digital studies is determining what level of support from study personnel is required to achieve an adequate level of participant adherence to study protocol (and therefore data completeness), with acknowledgment that some participants will require more assistance than others.

### Limitations

Narrative interviews with participants at the conclusion of previous studies, although not integrated into this study’s design, indicate that the ability to self-track activity using a smartwatch can be inherently motivating, at least for certain participants [5]. We did not integrate the results into in-person visits for clinical care, which has also been shown to increase adherence, although the frequency of visits can affect the regularity of data submission [12].

Adherence is higher in studies where the main aim is the use of a wearable device for healthy behavior change (ie, increased activity) [3,24,25], and the optimization of adherence with digital behavior change interventions has been detailed elsewhere [26]. We specifically designed this study to avoid such an intervention because we wanted to evaluate the role of reminders and centralized (remote) study coordinator communication on adherence to data submission. Whether incorporating an aim expected to increase the health of participants, along with the run-in period and reminder system of this study, would increase adherence further requires subsequent study. Although useful for identifying participants who would be motivated to complete the main study, the run-in period limited our ability to model for factors related to adherence in a more general population owing to our participant sample. For example, there were greater proportions of participants in this study who were White, female, and employed compared with patients in the Rheumatology Informatics System for Effectiveness registry, a large electronic health record database of people living with RA in the United States [27,28]. Moreover, there was a potential for selection bias because only RA patient members of the ArthritisPower registry with an email address could be invited to participate in the study. Finally, a review of retention indicators in remote digital studies asserted that the 2 most important factors extending retention were referral by a clinician to the study (increase of 40 days in median retention time) and compensation for participation (increase of 22 days) [29]. The study reported here included compensation but no clinician referral; therefore, combining both of these elements with other features unique to the study design of this study is also a topic for future research. Programs such as ours that use a smartphone device, with or without a biosensor, became reimbursable by insurance in 2022 (for in-app–only data collection) and 2019 (when incorporating a biosensor) by the Center for Medicare and Medicaid Services and other insurance plans [30,31]. The programs, termed Remote Therapeutic Monitoring and Remote Physiologic Monitoring provide opportunities to study the impact of data capture triggered by clinician referrals in nonresearch settings. The results of this study suggest that getting participants over the hurdles of the initial device setup and ePRO data collection can be successfully overcome in the first 1 to 2 days of digital health programs such as ours.

### Conclusions

Engaging patients in digital studies to adhere to a study protocol is a challenge that merits further examination to continue to understand and formulate best practices and guide future studies. Real-world evidence studies involving passive data collection in RA require participant-centric implementation and design to minimize the participant burden, promote longitudinal engagement, and maximize adherence. Passive data capture via activity trackers such as smartwatches, along with regular contact such as automated reminders and remote contact with study personnel, may facilitate greater participant adherence in providing longitudinal data for clinical trials and real-world studies.

## Appendix

Multimedia Appendix 1 Hierarchy of rules or triggers for participant contact.

Multimedia Appendix 2 Timing of participant contact by automated notifications and study coordinator.

Multimedia Appendix 3 Twilio automated messaging descriptions.

Multimedia Appendix 4 Study coordinator support for participant adherence.

## Abbreviations

BYOD: bring-your-own device
DIGITAL: Digital Tracking of Rheumatoid Arthritis Longitudinally
ePRO: electronic patient-reported outcome
LASSO: Least Absolute Shrinkage and Selection Operator
OR: odds ratio
PARADE: Patient Rheumatoid Arthritis Data From the Real World
PROMIS: Patient-Reported Outcomes Measurement Information System
RA: rheumatoid arthritis
RMD: rheumatic and musculoskeletal disease
