# Supplementary material for: Participant Engagement and Adherence to Providing Smartwatch and Patient-Reported Outcome Data: Digital Tracking of Rheumatoid Arthritis Longitudinally (DIGITAL) Real-World Study
Source: JMIR Hum Factors. 2023 Nov 7;10:e44034. doi: 10.2196/44034 (PMC10664008; doi:10.2196/44034)
Supplement: Multimedia Appendix 1 [file humanfactors_v10i1e44034_app1.docx]

Appendix Table 1. Hierarchy of Rules/Triggers for Participant Contact

| **Behavior** | **Hierarchy** | **Description** | **Action*** |
| --- | --- | --- | --- |
| SYNC | 1 | No Sync ≥6 days | Phone call |
| WEAR | 2 | Smartwatch wear time for 0 minutes ≥6 days | Phone call |
| WEAR | 3 | Smartwatch wear time for 1-800 minutes ≥6 days | Phone call |
| SLEEP | 4 | No sleep minutes ≥6 days | Phone call |
| SLEEP | 5 | Smartwatch wear time <1200 min + No sleep min ≥6 days | Phone call |
| wPRO | 6 | wPROs past due ≥6 days | Phone call |
| dPRO | 7 | dPROs past due ≥6 days | Phone call |
| WEAR | 8 | Smartwatch wear time for 0 minutes for 5 days | Phone call |
| WEAR | 9 | Smartwatch wear time for 1-800 minutes for 5 days | Phone call |
| SLEEP | 10 | No sleep minutes for 5 days | Phone call |
| SLEEP | 11 | Smartwatch wear time <1200 min + No sleep min for 5 days | Phone call |
| SYNC | 12 | No Sync 5 days | Text message |
| wPRO | 13 | wPROs past due 5 days | Text message |
| dPRO | 14 | dPROs past due 5 days | Text message |
| SYNC | 15 | No Sync 4 days | Text message |
| WEAR | 16 | Smartwatch wear time for 0 minutes for 4 days | Text message |
| WEAR | 17 | Smartwatch wear time for 1-800 minutes for 4 days | Text message |
| SLEEP | 18 | No sleep minutes for 4 days | Text message |
| SLEEP | 19 | Smartwatch wear time <1200 min + No sleep min for 4 days | Text message |
| wPRO | 20 | wPROs past due 4 days | Auto email/LSN |
| dPRO | 21 | dPROs past due 4 days | Text message |
| SYNC | 22 | No Sync 3 days | Auto email/LSN |
| WEAR | 23 | Smartwatch wear time for 0 minutes for 3 days | Text message |
| WEAR | 24 | Smartwatch wear time for 1-800 minutes for 3 days | Text message |
| SLEEP | 25 | No sleep minutes for 3 days | Text message |
| SLEEP | 26 | Smartwatch wear time <1200 min + No sleep min for 3 days | Text message |
| wPRO | 27 | wPROs past due 3 days | Auto email/LSN |
| dPRO | 28 | dPROs past due 3 days | Text message |
| Abbreviations: dPRO, daily patient-reported outcome; LSN, lock screen notification; wPRO, weekly patient-reported outcome | | | |

*Study team assumed that a phone call to a participant from Study Coordinator was the highest level of intervention escalation when urgent action was needed to minimize missing data.
