# Supplementary material for: Participant Engagement and Adherence to Providing Smartwatch and Patient-Reported Outcome Data: Digital Tracking of Rheumatoid Arthritis Longitudinally (DIGITAL) Real-World Study
Source: JMIR Hum Factors. 2023 Nov 7;10:e44034. doi: 10.2196/44034 (PMC10664008; doi:10.2196/44034)
Supplement: Multimedia Appendix 2 [file humanfactors_v10i1e44034_app2.docx]

Appendix Table 2. Twilio Automated Messaging Descriptions

| Issues | SMS template | Short form | Long form |
| --- | --- | --- | --- |
| No Sync 5 days | Message 1 | NoSync5 | ArthritisPower: Hi <first name>, it’s time to **sync your smartwatch**. Please open your Fitbit app and swipe down to sync your smartwatch. |
| wPROs 5 days | Message 2 | wPROs5 | ArthritisPower: Hi <first name>, please complete your **weekly** questions in the ArthritisPower app **if you haven't already done so. Here's the link https://arthritispower.org/app/#/home** |
| dPROs 5 days | Message 3 | dPROs5 | ArthritisPower: Hi <first name>, please complete your **daily** questions in the ArthritisPower app**if you haven't already done so**.  Here's the link https://arthritispower.org/app/#/home |
| No Sync 4 days | Message 4 | NoSync4 | ArthritisPower: Hi <first name>, it’s time to **sync your smartwatch**. Please open your Fitbit app and swipe down to sync your smartwatch. |
| Wear 0 min 4 days | Message 5 | NoWear4 | ArthritisPower: Hi <first name>, please remember **to wear your smartwatch today**. |
| Wear 1-800 min 4 days | Message 6 | LowWear4 | ArthritisPower: Hi <first name>, remember **to wear your smartwatch throughout the entire day!** |
| No sleep 4 days | Message 7 | NoSleep4 | ArthritisPower: **Sleep reminder** - Hi <first name>, please remember to sleep with your smartwatch on tonight. |
| Wear <1200 + No sleep 4 days | Message 8 | LowWearNoSleep4 | ArthritisPower: **Sleep reminder** - Hi <first name>, please remember to wear your smartwatch throughout the entire day and while you sleep. |
| wPROs 4 days | AUTO | wPROs4 | ArthritisPower: Hi <first name>, please complete your **weekly** questions in the ArthritisPower app **if you haven't already done so.  Here's the link https://arthritispower.org/app/#/home** |
| dPROs 4 days | Message 10 | dPROs4 | ArthritisPower: Hi <first name>, please complete your **daily** questions in the ArthritisPower app**if you haven't already done so**.  Here's the link https://arthritispower.org/app/#/home |
| No Sync 3 days | AUTO | NoSync3 | AUTO |
| Wear 0 min 3 days | Message 12 | NoWear4 | ArthritisPower: Hi <first name>, please remember to **wear your smartwatch today.** |
| Wear 1-800 min 3 days | Message 13 | LowWear4 | ArthritisPower: Hi <first name>, remember to **wear your smartwatch throughout the entire day!** |
| No sleep 3 days | Message 14 | NoSleep3 | ArthritisPower: **Sleep reminder** - Hi <first name>, please remember to sleep with your smartwatch on tonight. |
| Wear <1200 + No sleep 3 days | Message 15 | LowWearNoSleep3 | ArthritisPower: **Sleep reminder** - Hi <first name>, please remember to wear your smartwatch throughout the entire day and while you sleep. |
| wPROs 3 days | AUTO | wPROs3 | AUTO |
| dPROs 3 days | Message 17 | dPROs3 | ArthritisPower: Hi <first name>, please complete your **daily** questions in the ArthritisPower app**if you haven't already done so**.  Here's the link https://arthritispower.org/app/#/home |
| No Sync 5 days | Message 1 | NoSync5 | ArthritisPower: Hi <first name>, it’s time to **sync your smartwatch**. Please open your Fitbit app and swipe down to sync your smartwatch. |
