# Supplementary material for: Participant Engagement and Adherence to Providing Smartwatch and Patient-Reported Outcome Data: Digital Tracking of Rheumatoid Arthritis Longitudinally (DIGITAL) Real-World Study
Source: JMIR Hum Factors. 2023 Nov 7;10:e44034. doi: 10.2196/44034 (PMC10664008; doi:10.2196/44034)
Supplement: Multimedia Appendix 3 [file humanfactors_v10i1e44034_app3.docx]

Appendix Table 3. Timing of Participant Contact by Automated Notifications and Study Coordinator

| Pt ID | **@3 days** | **4 d** | **5 d** | **6d** | **7d** |
| --- | --- | --- | --- | --- | --- |
| **Sync** | Auto email & LSN | Auto email & LSN  Text message | Auto email & LSN  Text message | Auto email & LSN  Phone call | Auto email & LSN  Phone call |
| **Wear** | Text message | Text message | Phone call | Phone call | Phone call |
| **Sleep** | Text message | Text message | Phone call | Phone call | Phone call |
| **dPRO** | Auto email & LSN  Text message | Auto email & LSN  Text message | Auto email & LSN  Text message | Auto email & LSN  Phone call | Auto email & LSN  Phone call |
| **wPRO** | Auto email & LSN | Auto email & LSN | Auto email & LSN  Text message | Auto email & LSN  Phone call | Auto email & LSN  Phone call |
| **Battery** | If “low” then Auto email & LSN | | | | |
| Abbreviations: dPRO, daily patient-reported outcome; LSN, lock screen notification; wPRO, weekly patient-reported outcome | | | | | |

* dPRO = daily Patient Reported Outcome; LSN = lock screen notification; wPRO = weekly Patient Reported Outcome;

Automated actions

- Automated email + lock screen notification
  - If no sync > 3 days, then every day until completed
  - If wPRO is past due >3 days
  - If battery level is < “low”
