# Supplementary material for: Participant Engagement and Adherence to Providing Smartwatch and Patient-Reported Outcome Data: Digital Tracking of Rheumatoid Arthritis Longitudinally (DIGITAL) Real-World Study
Source: JMIR Hum Factors. 2023 Nov 7;10:e44034. doi: 10.2196/44034 (PMC10664008; doi:10.2196/44034)
Supplement: Multimedia Appendix 4 [file humanfactors_v10i1e44034_app4.docx]

Appendix Table 4: Study Coordinator Support for Participant Adherence

|  | Daily ePROs | Weekly ePROs | Smartwatch Sync | Sleep |
| --- | --- | --- | --- | --- |
| # Participants with Issue During Main Study Period^*1^ | 126 ( 45.3) | 205 ( 73.74 ) | 213 ( 76.62 ) | 206 ( 74.10 ) |
| # of Issues*** during Main Study period^*2^ | 428 | 571 | 792 | 912 |
| Resolved, n(%)^*3^ | 379 ( 88.55 ) | 528 ( 92.47 ) | 743 ( 93.81 ) | 798 ( 87.50 ) |
| Average # days to resolve issue, among issues that were resolved^*4^ | 3.02 | 4.28 | 2.37 | 3.28 |
| Custom SMS texts sent by case managers on unique calendar days^*5^ (Text – Manual. Unique calendar days in each column) | 225 | 82 | 188 | 265 |
| Phone call initiated on unique calendar days (hierarchical) ^*6^     Connected, spoke with participant (Call - Answered)     Connected, left VM or spoke with someone other than participant (Call - Voice Mail)      Never connected but working (Call - No Answer )     Phone line not working (Call – Disconnected) | 200 (100%)  46 (23.0%)  131 (65.5%)  12 (6.0%)  11 (5.5%) | | | |
| Never Resolved^*7^ | 49 (11.45) | 43 (7.53) | 49 (6.19) | 114 (12.5) |

After limiting to main study period and study participant identification, numbers counted Study Coordinator texts or phone calls on unique calendar days per footnotes.

*1. Percentage out of total 278 final IDs.

*2. Unique issue defined as same kind of issue.

*3. If the issue didn’t come up continuously, treated it as resolved. The percentage if out of total issue number.

*4. Issue resolved day is the total days of issue last continuously. This is conditional on the issue being resolved, those never resolved are not included in this row.

*5. This is DISPOSITION =”Text – Manual”, count unique calendar days on each column and patient.

*6. Following the hierarchical order in the table, count unique calendar days per patient.

*7. Not Resolved defined as year of snooze date on the issue is 2030, or if the issue record occurring on the last day of the study for that patient.
